# Mating behaviour in the sea slug *Elysia timida *(Opisthobranchia, Sacoglossa): hypodermic injection, sperm transfer and balanced reciprocity

**DOI:** 10.1186/1742-9994-4-17

**Published:** 2007-07-04

**Authors:** Valerie Schmitt, Nils Anthes, Nico K Michiels

**Affiliations:** 1 Evolutionary Biology, University Muenster, Huefferstrasse 1, 49149 Muenster, Germany; 2 Zoological Institute, Animal Evolutionary Ecology, Auf der Morgenstelle 28, 72076 Tuebingen, Germany

## Abstract

**Background:**

In simultaneous hermaphrodites with copulation and internal fertilization it is often unclear whether reciprocal sperm exchange results from the unconditional willingness of both partners to donate and receive sperm, or whether it follows from a more controlled process such as conditional reciprocal sperm exchange, i.e. sperm trading. While in some sea slugs mating is assumed to be based on sperm trading, it seems to be unconditional in others. Here, we describe the unusual mating behaviour of *Elysia timida*, a small sacoglossan, focussing on indications for conditional reciprocity.

**Results:**

*E. timida *shows an as yet unique combination of a long series of hypodermic transfers followed by a short phase with standard insemination into a female genital aperture. Hypodermic transfer takes place in the form of repeated small injections into the dorsal surface of the partner, interrupted by synchronised circling movements. In the final mating phase sperm is transferred into the female genital aperture in a short period. In both phases the two mating individuals show a high degree of transfer symmetry and synchrony. While total duration and number of transfers were balanced within pairs, they varied significantly between pairs. Furthermore, looking at individual hypodermic transfers within pairs, reciprocal transfers lasted longer than unilateral transfers. Final sperm transfers were always reciprocal except for two cases which also diverted from the usual pattern in ways that were suggestive of a conflict over reciprocity.

**Conclusion:**

Our results suggest that individual mating decisions in *E. timida *depend on what the partner does, indicating conditional reciprocity. If hypodermic transfers also involve the transfer of sperm (which remains to be confirmed), this system represents an up to now unique transition stage between hypodermic and standard insemination, both of which are widespread in this group of sea slugs, but never have been observed to co-occur within the same species.

## Background

### Mating conflicts in hermaphrodites

*Ad hoc *flexibility in sex roles and sex allocation is unique to simultaneous hermaphrodites. Possessing two sexes, however, potentially generates conflicts of interests between mating partners since both might prefer to mate either in the male role or in the female role [1-9]. This may in turn lead to different degrees of interest compatibility [9,10].

In gonochoric species, male reproductive success is typically limited by access to unfertilised eggs while female reproductive success is limited by resources to produce eggs (Bateman's principle). This principle is assumed to apply likewise to male and female functions in hermaphrodites [1,11,12]. If the reproductive success of the female function is limited by the availability of resources for egg production and not by the availability of allosperm (sperm received from a donor), this may lead to a conflict of interest between the sperm donor and the sperm receiver regarding the use of sperm [1]. For example, most if not all received sperm might be digested rather than be used for fertilization [10]. In addition, if individuals mate repeatedly as a female before egg fertilization, sperm from different donors will compete for fertilization [6,12-15]. Under these conditions, individuals should on the one hand persuade their partners to mate with them, but on the other hand suppress their willingness to remate later, adding to the mating conflict.

Repeated mating and sperm competition in combination with sperm digestion have been predicted to lead to increased male investment [16], at least under certain conditions [17]. The resulting high costs associated with sperm donation may explain why many internally fertilising hermaphrodites apparently hesitate to donate sperm and prefer mutual insemination [18]. Conditional reciprocity of sperm transfer, i.e. sperm trading, was first described for the opisthobranch *Navanax inermis *[19,20]. In *N. inermis *mating partners repeatedly alternate the female and the male role [19,20]. This results in balanced overall durations of sperm transfer between partners during a mating sequence, consistent with sperm trading [21]. The only experimental evidence for sperm trading thus far has been obtained from the sea slug *Chelidonura hirundinina *by controlling a slugs' ability to donate sperm without affecting other aspects of mating behaviour [22]. Here, focal animals more rapidly abandoned partners that did not transfer sperm.

Observing bilateral insemination does not suffice to infer conditionality since reciprocity can also result from a mutual, but unconditional willingness to donate sperm [5]. Several studies concluded against sperm trading despite explicit reciprocal insemination [23-25]. In a study of the snail *Lymnaea stagnalis *reciprocal sex role alternation only occurred when both mating partners had been isolated, which led the authors to question conditional reciprocity as a *per se *explanation for reciprocal mating patterns [26]. In addition, body size influences insemination durations and the degree of reciprocity in at least three different sea slug species [15,27,28] which makes the question of conditional reciprocity even more complicated.

The present study first describes the as yet unique mating behaviour of *Elysia timida*. We then examine whether exchange between partners is balanced and if there are indications for conditional reciprocity.

### Mating behaviour in opisthobranchs

Opisthobranch sea slugs are simultaneous hermaphrodites and possess complex reproductive systems for internal cross-fertilization [29-33]. Allosperm resorption has been shown to occur in several species [29,34] and can be assumed to be widespread due to the presence of a gametolytic gland in most groups [31]. Sea slugs typically donate and receive sperm reciprocally in a head-to-tail cross position. Partners face in opposite directions with their genital apertures on the front right body sides opposed and their penes mutually inserted [31,35]. In addition to this standard insemination mode a variety of alternatives exist. Some species form mating chains [31,35-37], alternate sex roles [19-22], or transfer sperm via externally attached spermatophores [31,38]. Hypodermic insemination, in which sperm is injected through the partner's body surface, is also widespread, particularly among the Sacoglossa [15,34,39-43]. Many species with hypodermic insemination have developed special penis armature, such as a sharp stylet [41]. In some species, sperm is injected through the body surface directly into the receptive organs while in others injection can take place anywhere into the partner's body [15,39-43]. Hypodermic insemination can be unilateral or bilateral in the same species [15,39,42]. Hypodermic insemination may also occur in species without penial armature [39,42,43]. Thus far, sea slugs have been observed to mate either by hypodermic injections or by standard insemination into the female aperture. The mating behaviour of *E. timida *is very unusual in that it combines standard insemination with a preceding phase of hypodermic injections, of which the function is as yet unclear. The only currently known example of such a combination stems from the gastropterid sea slug *Siphopteron quadrispinosum*, which uses a bipartite penis to hypodermically inject prostate secretions using its stylet-bearing branch before using its other branch for regular insemination [44].

### Study organism

*E. timida *is a small (< 2 cm) sea slug (Sacoglossa, Elysiidae) that lives in shallow, protected Mediterranean bays on rock surfaces that are covered with its food alga *Acetabularia acetabulum *[45-47]. Animals are opaque white with green lateral stripes and a mostly green colouration of the parapodia's upper surfaces stemming from endosymbiontic chloroplasts. Head, pericardial prominence and the parapodia's lower sides show a variable pattern of bright red spots, which allows individual recognition. *E. timida *has spatially separated male and female genital openings. The penis is positioned on the right side of the head, directly underneath the right eye. It is transparent and elongated during mating, allowing observation of sperm or fluid transfer with a stereo-microscope. The female aperture is located at the frontal base of the right parapodium. Transfers are predominantly reciprocal, sometimes unilateral.

## Results

### Description of mating sequences

**Figure 1:**
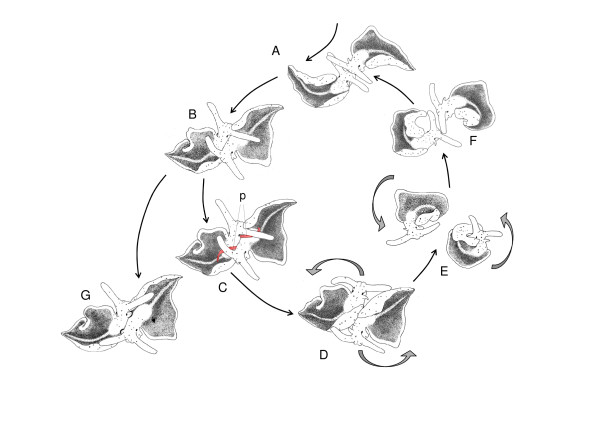
**Mating sequence of *E. timida***. **A **Head-to-head contact **B **Copulation position **C **Hypodermic transfer, showing extended penises (*p*, marked red) **D **Start of circling **E **Circling **F **End of circling **G **Sperm transfer into female aperture. Drawings by V. Schmitt from video recordings.

**Figure 2:**
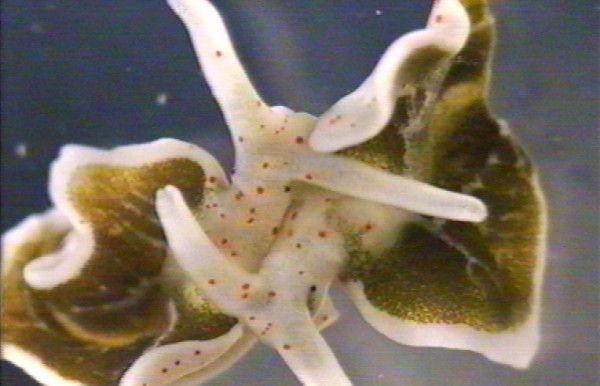
**Two *E. timida *individuals in copulation position**. (from video recording).

The observed *E. timida *individuals initiated mating after meeting head-to-head (N = 24 pairs). In one additional pair, individuals met sideways, but instantly moved into a head-to-head position. Subsequently, individuals stopped gliding and touched each other with their head and elongated rhinophores (Fig. 1A). These first contacts lasted 21.1 ± 33.2 *s *(range: 1 – 155 *s*, N = 25, data per pair). From this position the two slugs bent their heads slightly to the left and slowly moved forward along the right side of the partner until reaching the base of the partner's right parapodium. In this way mating partners took up the typical head-to-tail mating position (Fig. 1B, Fig. 2)

Twenty-two out of 25 pairs started penis protrusion immediately and simultaneously during initial head-to-head contact or first copulation position. The remaining pairs everted their penis soon after initial contact. In twenty pairs both partners kept their penis everted throughout the complete mating sequence.

**Figure 3:**
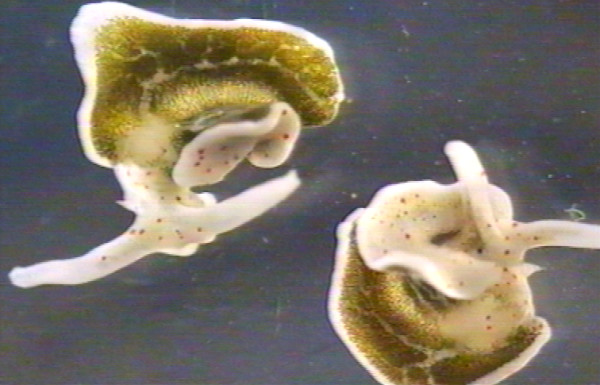
**Mating *E. timida *individuals performing the stereotypic anticlockwise circling movement**. (from video recording).

When partners had taken up copulation position, they either stayed in position for a hypodermic transfer attempt (Fig. 1C, Video 2) or they started to circle (Fig. 1D, Fig. 3, see below).

If starting a hypodermic transfer attempt, partners stretched their penis up to one third of their body length to reach the dorsal surface of their partner (Fig. 1C). The movable penis tip touched the partner's dorsal surface in an attempt to insert. In 25.9% of insertion attempts the penis transmitted a white substance on or into the dorsal surface of the partner. Substance transfer by hypodermic injection lasted from 3 *s *to 32.25 min (x¯
 MathType@MTEF@5@5@+=feaafiart1ev1aaatCvAUfKttLearuWrP9MDH5MBPbIqV92AaeXatLxBI9gBaebbnrfifHhDYfgasaacH8akY=wiFfYdH8Gipec8Eeeu0xXdbba9frFj0=OqFfea0dXdd9vqai=hGuQ8kuc9pgc9s8qqaq=dirpe0xb9q8qiLsFr0=vr0=vr0dc8meaabaqaciaacaGaaeqabaqabeGadaaakeaacuWG4baEgaqeaaaa@2E3D@ = 6.47 ± 7.63 min, N = 44 individuals). Several such hypodermic transfers occurred per mating sequence (x¯
 MathType@MTEF@5@5@+=feaafiart1ev1aaatCvAUfKttLearuWrP9MDH5MBPbIqV92AaeXatLxBI9gBaebbnrfifHhDYfgasaacH8akY=wiFfYdH8Gipec8Eeeu0xXdbba9frFj0=OqFfea0dXdd9vqai=hGuQ8kuc9pgc9s8qqaq=dirpe0xb9q8qiLsFr0=vr0=vr0dc8meaabaqaciaacaGaaeqabaqabeGadaaakeaacuWG4baEgaqeaaaa@2E3D@ = 2.68 ± 2.30, range: 0 – 9, N = 50 individuals).

After each hypodermic injection the penis was withdrawn from the partner (but as a rule not fully retracted) and individuals started a synchronous, stereotypic circling movement in a counter-clockwise direction (Fig. 1D, Fig. 3, Video 1). If only one individual started circling, its partner usually responded by circling as well (Fig. 1E and Fig. 3, Video 1). Most (81%) circling phases involved a single circle only (1.06 ± 3.04 min, N = 50 individuals). In the other cases individuals kept circling, in one case up to 40.8 min. After resuming copulation position, a new hypodermic transfer attempt was started (Fig. 1B–C). Transfer attempts and circling movements were alternated 1 to 34 times during a single mating sequence (x¯
 MathType@MTEF@5@5@+=feaafiart1ev1aaatCvAUfKttLearuWrP9MDH5MBPbIqV92AaeXatLxBI9gBaebbnrfifHhDYfgasaacH8akY=wiFfYdH8Gipec8Eeeu0xXdbba9frFj0=OqFfea0dXdd9vqai=hGuQ8kuc9pgc9s8qqaq=dirpe0xb9q8qiLsFr0=vr0=vr0dc8meaabaqaciaacaGaaeqabaqabeGadaaakeaacuWG4baEgaqeaaaa@2E3D@ = 12.7 ± 6.6, N = 25 pairs).

The hypodermic injection and circling phase described above represented the longer part of the mating sequence. It was followed by a short final phase in 21 out of 25 matings. Here, individuals took up copulation position again, but inserted their penis into the partner's female aperture (Fig. 1G, Video 3). Mates inserted mutually and almost simultaneously and transferred sperm in a short time period (x¯
 MathType@MTEF@5@5@+=feaafiart1ev1aaatCvAUfKttLearuWrP9MDH5MBPbIqV92AaeXatLxBI9gBaebbnrfifHhDYfgasaacH8akY=wiFfYdH8Gipec8Eeeu0xXdbba9frFj0=OqFfea0dXdd9vqai=hGuQ8kuc9pgc9s8qqaq=dirpe0xb9q8qiLsFr0=vr0=vr0dc8meaabaqaciaacaGaaeqabaqabeGadaaakeaacuWG4baEgaqeaaaa@2E3D@ = 1.61 ± 0.75 min, range: 0.57 – 4.12 min, measurements available for N = 41 individuals). Out of the 21 matings with a final insemination phase 20 mating sequences ended with a reciprocal final insemination. Only one single mating ended with a unilateral final insemination.

After reciprocal final sperm transfer slugs withdrew and separated simultaneously, gliding off into opposite directions. Complete mating sequences lasted 42.9 ± 14.7 min (range: 10.8 to 79.5 min, N = 25). After insemination and separation a white blob of semen remained attached to the outside of the female aperture. Investigation under a microscope revealed that sperm had a simple thread-like shape without a prominent head and showed vigorous spiralling movement of the middle section. In structure and movement the sperm of *E. timida *correspond strongly to that described for *E. patina *[42].

### Hypodermic injections

**Figure 4:**
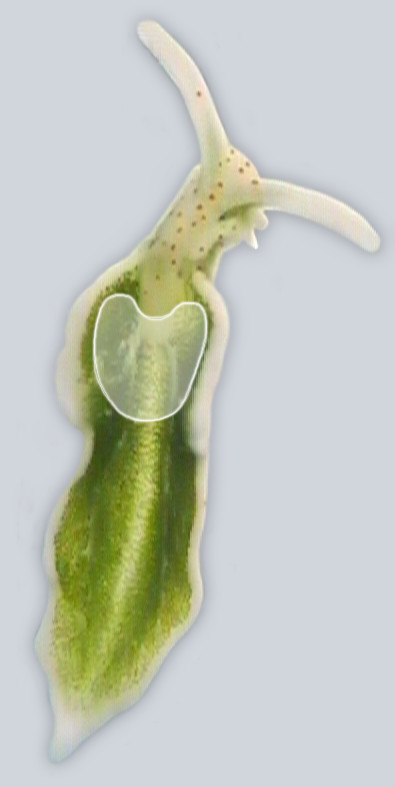
**The main area of hypodermic transfers in the dorsal notum**. (highlighted).

The main injection area was the anterior part of the partner's inner parapodial dorsal surface including the posterior part of the pericardial prominence and its surroundings up to the frontal third of the dorsal surface (Fig. 4). In 123 out of 132 cases transfer by hypodermic injection occurred in this area. In four cases transfer region was the partner's head and in another four the anterior lateral side of the partner's right parapodium. The substance transferred by hypodermic injections could not be isolated as it was either injected directly or dispersed into the water.

Although *E. timida *shows hypodermic injection, no special penis armature in the form of a stylet or hook(s) was found on inspection with SEM and stereo-microscopy. One animal that had received four hypodermic transfers notum was fixed 5 *h *after copulation. On inspection with SEM four bumps were identified in the main insertion area. An individual kept in isolation had a very smooth notum without bumps. These observations confirm our assumption that hypodermic injection results in the transfer of a substance.

### Reciprocity

**Figure 5:**
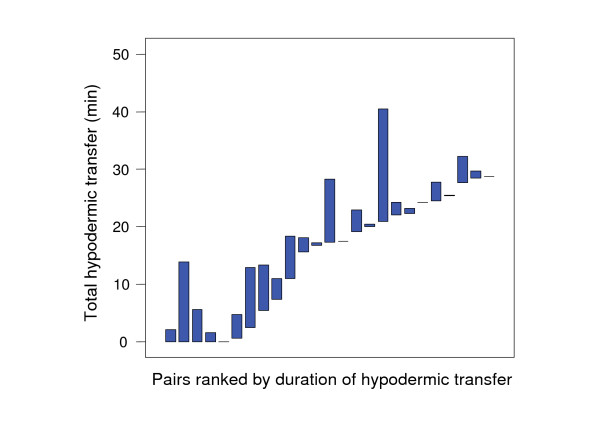
**Range plot of total hypodermic transfer duration per mating for both partners (represented by the minimum and maximum) for 25 pairs, ranked by the minimum value per pair**. Dashes indicate pairs with identical values for both partners. There is a high degree of within-pair similarity in the duration of hypodermic transfer.

**Figure 6:**
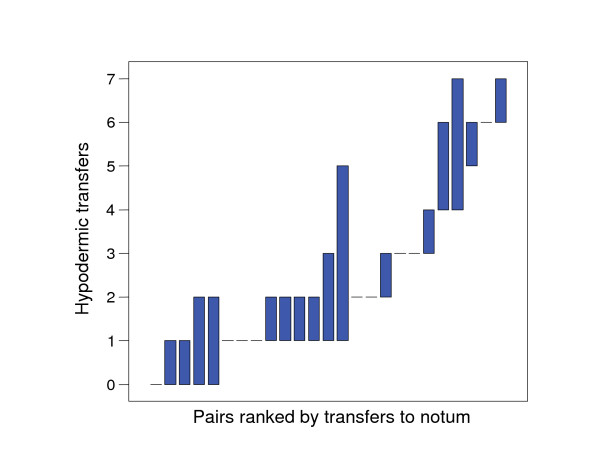
**Range plot of number of hypodermic transfers per mating for both partners (represented by the minimum and maximum) for 25 pairs, ranked by the minimum value per pair**. Dashes indicate pairs with identical values for both partners. There is a high degree of within-pair similarity in the number of hypodermic transfers.

Fifty-two out of 80 hypodermic transfers were performed reciprocally. Although not all hypodermic transfers were reciprocal, the total duration of all hypodermic transfers during a mating session was balanced between mates but significantly different between pairs (Kruskal-Wallis *χ*^2 ^= 46.4, *d.f*. = 24, *P *= 0.004; Fig. 5; see methods for rationale behind this analysis). The same applies to the number of hypodermic transfers (Kruskal-Wallis *χ*^2 ^= 42.7, *d.f*. = 24, *P *= 0.011; Fig. 6). Direct observation suggested that individuals transferred fluid at a constant rate; therefore the amount received should be similar to the amount donated.

**Figure 7:**
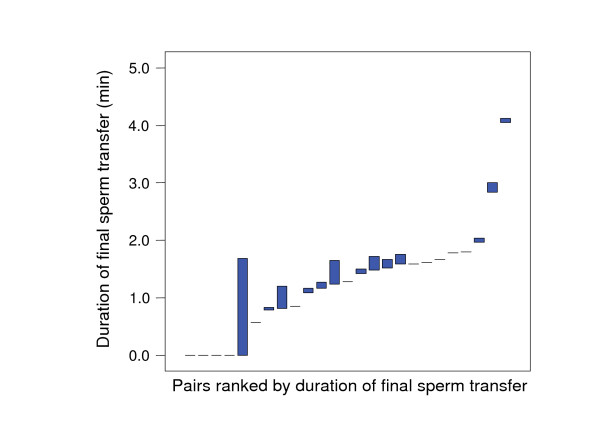
**Range plot of the duration of sperm transfer into the female aperture per partner (represented by the minimum and maximum) for 25 pairs, ranked by the minimum value per pair**. Dashes indicate pairs with identical values for both partners. There is a high degree of within-pair similarity in the duration of sperm transfer.

The duration of final sperm transfer was also balanced between partners but differed significantly between pairs (Kruskal-Wallis *χ*^2 ^= 45.1, *d.f*. = 24, *P *= 0.006; Fig. 7), coinciding with the synchrony with which these transfers were performed by both partners. The four matings in which partners did not transfer sperm into the female aperture occurred in pairs in which only one partner had transferred hypodermically while the other had not.

### Conditional reciprocity?

**Figure 8:**
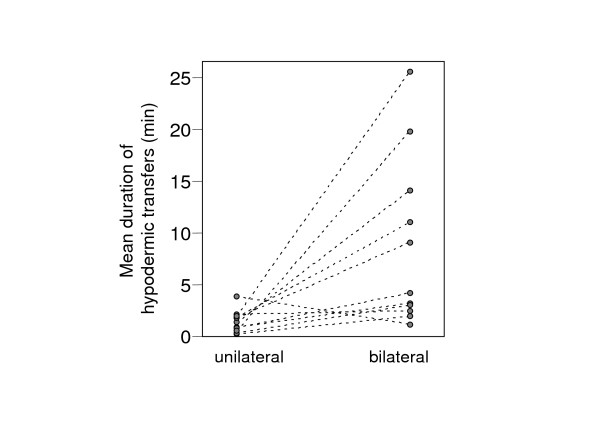
**Average durations of single unilateral and bilateral hypodermic transfers per mating in 11 individuals (see text)**. Individuals transferred clearly longer by bilateral than by unilateral transfers.

As already indicated by within-pair symmetry in total injection duration, individuals performed longer (or shorter) single hypodermic injections when they received more (or less) from their partner. To illustrate this effect, unilateral and reciprocal hypodermic transfers were compared within those 11 mating sequences where at least one partner performed both a unilateral and a bilateral transfer. From each of those pairs only one partner was considered. In cases where both partners had at least one unilateral transfer, we always used the individual with the longest unilateral donation to obtain a conservative estimate. We then compared how long these individuals donated hypodermically by unilateral versus bilateral transfers during a mating sequence (Fig. 8). In all but one case the average duration of single bilateral hypodermic transfers per individual during a mating sequence was higher than the average duration of single unilateral transfers (Wilcoxon Signed Ranks Test, *z *= -2.49, *P *= 0.013). This suggests a preference for reciprocal transfers as well as the presence of a control mechanism to check what the partner does.

There are also possible indications for conditional reciprocity in the final mating phase. Individuals often touched the partner's female aperture with their penis several times before final sperm transfer and seemed to "wait" for the partner to react reciprocally. Final sperm transfer was reciprocal and approximately simultaneous except for two cases. Here, only one partner transferred sperm, which was followed by an exceptionally long circling phase of about 30 min. This is in contrast to the immediate separation after reciprocal sperm transfer. In one of these two cases, partners finally transferred reciprocally and then separated immediately. Additionally, both pairs displayed repeated, alternating penis retractions and protrusions while circling, which was never the case in other matings. This aberrant behaviour suggests a conflict over failed reciprocity.

## Discussion

### Penial armature

Although *E. timida *performs hypodermic injections, special penial armature could not be detected externally. In a comparative study on penial armature in different sacoglossans it has been reported that penial stylets – when not in use – are completely housed in a pocket at the tip of the penis [41]. Although a penis stylet is common among sacoglossans, it is considered an apomorphy [48] and to be absent from species of the genus *Elysia *[45,48-50]. At least three species of *Elysia *perform exclusively hypodermic insemination despite the fact that their penis is unarmed [39,42,43]. The only exception is *E. patina*, which possesses a very small and thin cuticular stylet at the tip of the penis and also inseminates hypodermically [42]. Given that we have never seen any indication of a stylet, we assume for now that the penis in *E. timida *is indeed unarmed as is typical for *Elysia *spp. It is not known how hypodermic transfer is achieved without the aid of penial armature. Possibilities considered are enzymatic attack [51] or turgor pressure [39].

### Why hypodermic transfers?

Assuming that the hypodermic transfers are functional and not a phylogenetic relic, we suppose that the injected substance induces an effect in the receiver that is in the interest of the sperm donor. For the time being, we must consider two alternatives regarding the composition of the fluid that is injected: (1) male prostate fluid only, or (2) semen, consisting of sperm and prostate fluid.

From the perspective of the sperm donor, prostate fluids can represent a manipulating or stimulating cocktail that changes the physiology of the receiver in the interest of the sperm donor [5,52-54]. In a pre-insemination context, the injections might serve to manipulate the partner to accept the less desirable sex role, as seems to be the case in the gastropterid sea slug *Siphopteron quadrispinosum *[44]. In *E. timida*, pre-insemination manipulation may be required as a part of a ritualised courtship aiming at synchrony and reciprocity. In a post-insemination context, prostate secretions can possibly influence sperm usage and increase the relative paternity of the sperm donor as is achieved by the love dart in Helicid snails [55,56]. Similar mechanisms are likely to exist in other hermaphrodites [5,33]. It is important to stress that – even if hypodermic injection may have evolved from a donor-specific benefit, we do not exclude that receivers may receive some form of benefit from being injected – as suggested by the "cooperative" and ritualised behaviour lacking signs of avoidance.

Hypodermic transfer may, however, also involve sperm transfer. Support for this comes from the observation that hypodermic insemination is widespread among sacoglossans [15,34,39-43], but transfer of fluid without sperm has never been reported. In *Alderia modesta *it was shown that hypodermic insemination anywhere in the body leads to successful egg fertilization [15]. The copulation position and insertion area described for *Elysiasubornata*, which inseminates exclusively hypodermically [42], are very similar to what we describe for the hypodermic transfers in *E. timida*. This similarity includes the position and appearance of the bumps seen after hypodermic injection. These observations lend further support to the possibility that *E. timida *is unique in that it combines standard with hypodermic sperm transfer. All the possibilities for stimulation and manipulation before and after sperm transfer discussed above remain in effect under this scenario.

### Conditional reciprocity

In opisthobranchs with one common genital papilla with both female and male apertures that are crosswise inserted, the anatomical conditions facilitate reciprocity. A separation of the male and female aperture, as in sacoglossans, may provide more scope for non-reciprocal behaviour as genital contact at one "meeting point" does not require genital contact at the other. Hypodermic copulation in *E. maoria *is reported to be sometimes reciprocal, sometimes non-reciprocal, which in non-reciprocal cases often means that both partners have extended their penis, but only one actually inserts and transfers [39]. This is very similar to the hypodermic transfers in *E. timida*. The hypodermic transfers in *E. patina *and *E. subornata *are also sometimes non-reciprocal [42] and the same applies to several other sacoglossans (e.g. [15,43]). Despite the possibility of unilateral transfers, *E. timida *showed extreme synchrony and reciprocity between mating partners, with much variation between pairs, but little within pairs. These findings suggest that mating in *E. timida *follows a form of conditional reciprocity. An experimental approach, such as used by [22,24] is required to prove this proposition.

If it is sperm that is injected hypodermically, it is important to stress that the pattern of sperm exchange is not based upon alternating insemination as in *Navanaxinermis *[21] or *Chelidonura hirundinina *[22], but on repeated, mostly simultaneous transfer. Simultaneous transfer is one possible type of sperm trading [18], but has never been described as a repeated process. Other unusual characteristics of *E. timida *mating behaviour such as slow movement and circling may facilitate reciprocity. Altogether the mating behaviour of *E. timida *is the exact opposite of that of the "hit-and-run" species in which mating individuals attempt to inseminate a partner quickly and unilaterally [57]. *E. timida *partners rather seem to "sit-and-wait" to assess the degree of synchrony and reciprocity they receive from their partner. A measure indicating this hesitance is the long duration of the whole mating sequence.

## Conclusion

The mating behaviour of *E. timida *represents a hitherto undescribed combination of repeated hypodermic injections and standard sperm transfer into a female genital aperture. If the hypodermic injections transfer sperm, this mating system could represent a transition between the two insemination types and a key system to analyse the evolution of insemination mechanisms.

Sexual behaviour was very synchronised and balanced between partners, leading to a high degree of reciprocity. The synchrony within pairs versus the differences between pairs indicates that mates adapt their behaviour in response to that of their partner. Hypodermic injections were significantly shorter when performed unilaterally than bilaterally. The final sperm transfers were always reciprocal except for only two non-reciprocal cases in which individuals reacted with aberrant behaviour indicating a conflict over non-reciprocity. Combined, our findings suggest that mating in *E. timida *is likely to be based on conditional reciprocity or sperm trading.

## Methods

### Collection and maintenance

*E. timida *was collected while SCUBA diving and snorkelling in 1–6 m depth at three sites in Banyuls sûr Mer, France, in spring 2003. Slugs were kept in a 160 × 60 × 15 cm basin with running sea water and an *ad libitum *supply of their food alga *Acetabularia acetabulum*. The basin was enriched with stones and other algae. It was exposed to natural sunlight through a window enabling animals to photosynthesize by means of their endosymbiontic chloroplasts. Individuals were kept for up to 48 h before observation. Each pair was observed only once in order to obtain independent data.

### Behavioural observations

For each observation session two individuals were paired in a 100 ml glass bowl and observed continuously through a stereo-microscope. By choosing a partner of approximately the same size, confounding effects resulting from size differences were minimized. Previously, size-differences between mating partners have been shown to influence mating behaviour in various opisthobranchs [14,15,28,58-62]. But since effects of relative size were not the main focus of this study, we decided to reduce potentially influencing factors by using size-assorted pairs as in [24].

Each pair was observed until partners had separated completely after a successful mating sequence. From the first five complete mating sequences an ethogram of the species specific mating behaviour was defined and a code for behavioural elements was developed. The occurrence and duration of these behavioural elements were recorded. Data were obtained for 25 complete mating sequences involving 50 different individuals.

### Scanning electron microscopy

For morphological examination, individuals were fixed with Bouin's solution and transferred after at least 24 hours in 70% ethanol. The fixed specimens were critical-point-dried with a Balzers CPD 020 critical-point drying apparatus, prepared with gold in a Leitz-contrasting device and scanned with a Hitachi S 530 scanning electron microscope.

### Statistics

All statistical analyses were performed using SPSS 10.0 and 11.5. Vreys & Michiels [63] discussed the difficulties of analysing correlations of two hermaphroditic partners by the application of regular correlation coefficients. Instead they used a one-way analysis of variance (ANOVA) which tests for similarity *within *pairs relative to *between *pairs [12,18,21]. Since the Levene test showed a significant deviation from homogeneity of variances for the variables that should be analysed, we used the Kruskal-Wallis test in this study to compare variance within pairs versus between pairs. Means are presented ± standard deviation with number of cases and range. Where necessary, data were averaged per pair prior to analysis to avoid pseudoreplication. Data of behavioural patterns varying in the two mating partners were analysed per individual and labelled as such.

## Authors' contributions

The present study was a part of VS's diploma project. VS carried out the behavioural studies in Banyuls sûr mer, performed the statistical analyses, and drafted the manuscript. NA carried out histological analyses and assisted in data interpretation and manuscript editing. NKM supervised the study, participated in its design and coordination and helped to analyze the data and draft the manuscript. All authors read and approved the final manuscript.

## Supplementary Material

Additional file 1 **Video 1: **Circling and copulation position. Click here for file

Additional file 2 **Video 2: **Hypodermic transfer. Click here for file

Additional file 3 **Video 3: **Final sperm transfer. Click here for file
